# HMGB1 and Cord Blood: Its Role as Immuno-Adjuvant Factor in Innate Immunity

**DOI:** 10.1371/journal.pone.0023766

**Published:** 2011-08-22

**Authors:** Alessandra Ciucci, Ida Gabriele, Zulema A. Percario, Elisabetta Affabris, Vittorio Colizzi, Giorgio Mancino

**Affiliations:** 1 Immunopathology Unit, Research Center, San Pietro Hospital, Fatebenefratelli, Rome, Italy; 2 Biology Department, University of Rome “RomaTre”, Rome, Italy; 3 Biology Department, University of Rome “Tor Vergata”, Rome, Italy; Centre de Recherche Public de la Santé (CRP-Santé), Luxembourg

## Abstract

In newborn the innate immune system provides essential protection during primary infections before the generation of an appropriate adaptive immune response that is initially not fully operative. Innate immune response is evoked and perpetuated by molecules derived from microorganisms or by the damage/death of host cells. These are collectively known as damage-associated molecular-pattern (DAMP) molecules. High-mobility group box 1 protein (HMGB1) or amphoterin, which previously was considered to be only a nuclear factor, has been recently identified as a DAMP molecule. When it is actively secreted by inflammatory cells or passively released from necrotic cells, HMGB1 mediates the response to infection, injury and inflammation, inducing dendritic cells maturation and T helper-1-cell responses. To characterize the role of HMGB1 in the innate and immature defense mechanisms in newborns, human cord blood (CB) mononuclear cells, in comparison to adult peripheral blood (PB) mononuclear cells, have been analyzed for its expression. By flow cytometry and western blot analysis, we observed that in CB and PB cells: i) HMGB1 is expressed on cell surface membranes of myeloid dendritic cell precursors, mostly, and lymphocytes (gamma/delta and CD4^+^ T cells) to a lesser extent; ii) different pro-inflammatory stimuli or molecules that mimic infection increased cell surface expression of HMGB1 as well as its secretion into extracellular environment; iii) the treatment with synthetic molecules such as aminobisphosphonates (ABs), identified to be γδ T cell antigens, triggered up-regulation of HMGB1 expression on mononuclear cells, as well γδ T lymphocytes, inducing its secretion. The modulation of its secretion and the HMGB1-mediated migration of monocytes indicated HMGB1 as regulator of immune response in an immature system, like CB, through engagement of γδ T lymphocytes and myeloid dendritic cell precursors, essential components of innate immunity. In addition, the increased HMGB1 expression/secretion triggered by ABs, previously characterized for their immuno-modulating and immune-adjuvant capabilities, indicated that immunomodulation might represent a new therapeutical approach for neonatal and adult pathologies.

## Introduction

The neonatal immune system is generally considered to be immature and less functional compared to adult counterpart. This immaturity is thought to account for the failure of the newborn to mount robust and protective response against several pathogens, resulting in increased mortality [1]–[4]. The impairment of the newborn immune system may result from the combined effects of a number of factors including: immaturity of its cellular components; lack of previous exposure to antigens; intra-uterine exposure to unique hormonal and cytokine environment which may support Th2 subset development; low proliferation capacity of T lymphocytes and its impaired Th1 cytokine production. Therefore, at the onset of microbial infections, before the generation of an appropriate adaptive (antibody or T cell mediated) immune response, the most important line of defense is innate immunity, where γδ T lymphocytes together with dendritic cells (DCs), macrophages/monocytes and NK cells are the essential components. Innate immunity triggers proinflammatory reactions and is involved in the initial clearance of pathogens.

During the last decade it has been observed that the innate immune response also orchestrates the subsequent adaptive immune response through cytokines and chemokines released by macrophages, DCs and Langerhans cells that are differently activated by the initial innate response. Unlike adaptive immunity, innate immunity is programmed to recognize series of molecular patterns present at the infected lesion: (i) the patterns that are presented by microorganisms [pathogen-associated molecular patterns (PAMPs)], and (ii) the patterns of host intracellular molecules secreted by dying host cells into the extracellular spaces upon microorganism-induced damage [damage-associated molecular patterns (DAMPs)] [5]–[8]. Consequently, the co-existence of PAMPs and DAMPs signals after invasion by pathogenic microorganisms are closely associated to tissue damage.

The list of DAMPs candidate molecules is getting longer and includes high mobility group box 1 (HMGB1), heat shock proteins, interleukin-1α (IL-1α), defensins, annexins, and S100 [9]–[13]. HMGB1, or amphoterin, previously has been reported to be only a nuclear factor able to enhance transcription. More recently, HMGB1 has been demonstrated to be a crucial cytokine that mediates the response to infection, injury and inflammation. HMGB1 is a 30 kD nuclear protein of 215 amino acids. It includes two DNA-binding domains: the A box and the B box, and a negatively charged C-terminal tail. Truncation of HMGB1 indicates that the recombinant A box (1–89) acts as a specific antagonist, whereas the cytokine activity of HMGB1 is determined by the recombinant B box (90–176) [14]. The first 20 amino acids of the recombinant B box represent the minimal peptide maintaining cytokine activity.

HMGB1 recruits inflammatory cells and activates innate immune cells. Further, after release from necrotic cells or its secretion by activated macrophages, it regulates adaptive immunity [13], [15]–[17]. Moreover, HMGB1 supports the maturation and migration of antigen-presenting cells, in particular DCs, to secondary lymphoid organs where these cells play a central role in the activation of naive T cells, in the promotion and induction of Th1 responses, and clonal expansion of antigen-specific T cells, the process at the basis of the adaptive immune response [18], [19]. Recently, Kalyan S [20] has reported that peripheral γδ T lymphocytes, previously activated by nonpeptidic antigen isopentylpyrophosphate (IPP), induced the upregulation of CD40 on monocytes and the local release of HMGB1, indicating γδ T cells as immune modulators of stress stimuli and Th1 polarization together with HMGB1. In immune response, γδ T cells represent the first line of defense and are considered to be the border between innate and adaptive immune response. Interestingly, aminobisphosphonates (ABs) which are synthetic compounds commonly used to treat bone disease and hypercalcemia in patients with multiple myeloma, breast or prostate cancer, have been identified also as antigens for γδ T cells, indicating these molecules as immunomodulating factors [21]–[26].

The expression of HMGB1 and its role in immune response has been demonstrated successfully in adult peripheral blood (PB). Only recently, Buhimashi CS *et al* [27] observed that HMGB1, together with soluble receptor for advanced glycation end-products (sRAGE) and S100, may be important mediators of cellular injury in fetuses and crucial factor in preterm birth induced inflammation. Therefore, due to the importance in understanding immature and innate immune profiles in newborn, we have characterized the expression and modulation of HMGB1 in human cord blood (CB) mononuclear cells.

## Results

### HMGB1 is expressed on cell-surface of human cord blood cells

**Figure 1:**
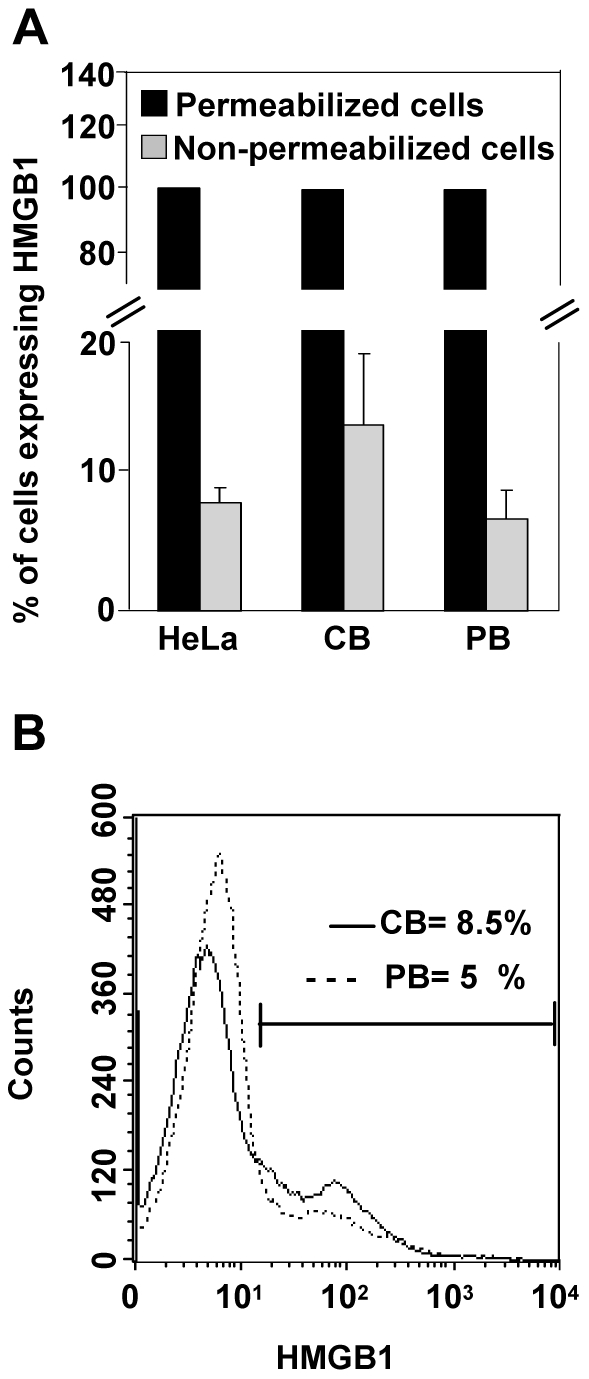
HMGB1 is expressed in human cord blood cells. Mononuclear cells isolated from human cord (CB) and peripheral blood (PB) have been cultured in complete growth medium in absence of external stimuli. (A) The intracellular and surface expression of HMGB1 has been determined 48 h after isolation by flow cytometry analysis in permeabilzed and non-permeabilized cells, respectively. HeLa cells represent the reference cell line known to be HMGB1 positive. Values (mean ± SD of eight experiments from different donors) are expressed as percentage of cells labeled with anti-HMGB1 antibody. (B) The fluorescence histogram plot derived from FACS analysis shows the HMGB1 expression profile of CB cells (solid line) and PB cells (dotted line). The percentage of HMGB1 positive cells is indicated in graph. The histogram plot is representative of eight different experiments.

Since HMGB1 is present in serum of human cord blood, indicating it as possible mediator of inflammation in fetuses [27], by FACS analysis we first evaluated the intracellular expression of HMGB1 in human CB and PB derived cells in comparison to HeLa cells, known to express HMGB1. Due to the fact that HMGB1 is a nuclear factor, the totality of CB and PB permeabilized cells showed the presence of intracellular HMGB1 expression at a comparable frequency of HeLa cells (Fig. 1A). In addition, we evaluated cell surface expression of HMGB1 in absence of permeabilization, ever since any data was reported on its expression in CB cells. To this end, mononuclear cells isolated from human CB were cultured in complete growth medium and the HMGB1 expressing cells were determined 48 h after isolation. Flow cytometry analysis indicated that 13%±4 (n = 8) CB mononuclear cells expressed HMGB1 on their surface whilst in PB cells HMGB1 was present on 6.5%±1.8 of cells (n = 8) (Fig. 1A). Interestingly, CB cells presented a significantly higher constitutive HMGB1 expression than PB (p = 0.02). Fig. 1B shows the overlay of fluorescence histogram plots in one representative CB respect to PB, indicating the higher percentage of HMGB1 expressing cells (8.5% in CB versus 5% in PB). In HeLa cells, HMGB1 is expressed by a mean of 7.6%±1 cells.

### HMGB1 is expressed mainly on myeloid DC precursors

**Figure 2:**
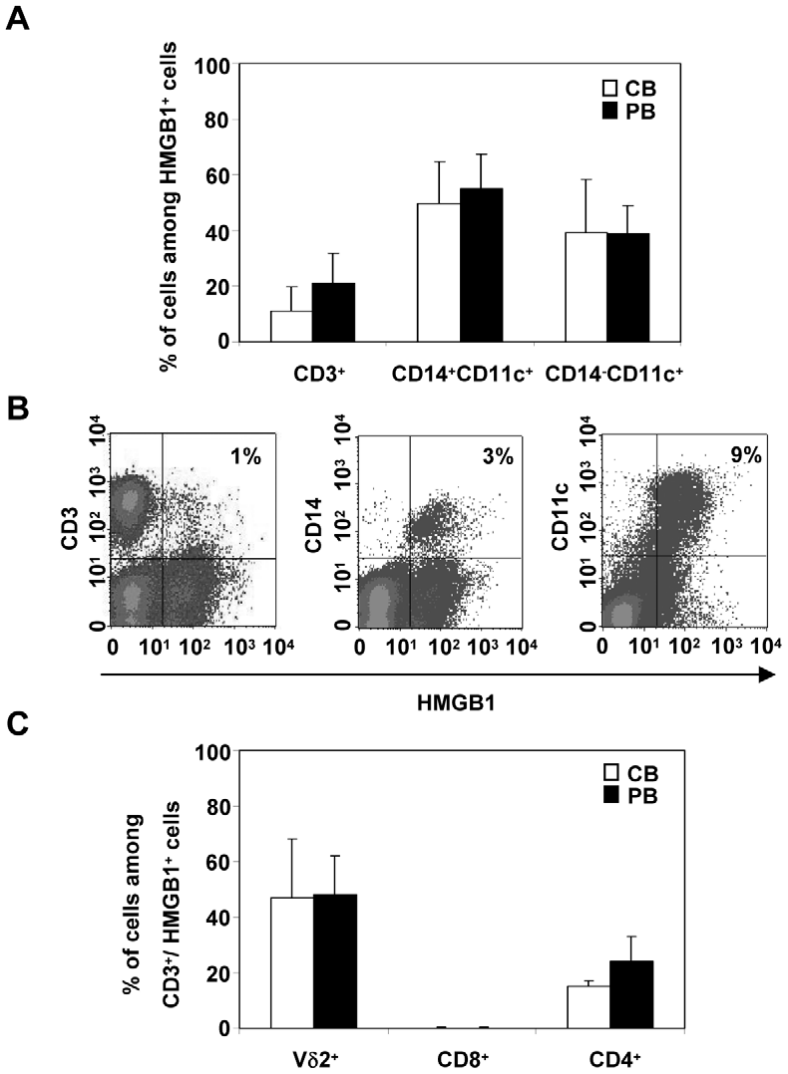
Distribution of HMGB1 cell surface expression: myeloid DC precursors are main cell subset. At 48 h after isolation of mononuclear cells from CB and PB, multi-color flow cytometric analysis has been performed to evaluate surface-expressed HMGB1 and expression of cellular differentiation markers. (A) Two main subsets were identified to be HMGB1 positive in CB and PB: CD3^+^, lymphocytes; CD14^+^CD11c^+^ and CD14^−^ CD11c^+^, myeloid DC precursors. Values (mean ± SD of five experiments from different donors) are expressed as percentage of HMGB1 positive cell subset among the totality of HMGB1 expressing cells. (B) Representative histogram plots derived from two-color FACS analysis show the percentage, indicated on the right of each plot (upper right panel), of HMGB1^+^ CD3^+^ (left plot), HMGB1^+^ CD14^+^ (middle plot) and HMGB1^+^ CD11c^+^ (right plot) in CB cells. Histograms plots are representative of five different experiments. (C) In order to characterize the different T lymphocyte subsets, CB and PB cells have been gated for lymphocytes and multi-color stained with HMGB1, CD3 and gammadelta (Vδ2) or CD8 or CD4 antibodies. The values are the mean ± SD of five experiments from different donors.

After 48 h of cell culture in complete growth medium, by multi color flow cytometric analysis we evaluated the cell distribution of HMGB1 expression on different cell subsets of CB and PB. As shown in Fig. 2A, in CB cells about 90% of HMGB1 is expressed on myeloid DC precursors identified in two subsets with CD14^+^CD11c^+^ and CD14^−^D11c^+^ phenotype [28]. Only a small portion of HMGB1 (11%±8) positive cells is represented by CD3^+^ subset. These results were further corroborated by data achieved in PB, showing a pattern of cell surface HMGB1 expression comparable to CB (Fig. 2A). In Fig. 2B, a representative dual-color FACS plot analysis indicated that the large part of CB CD11c^+^ cells were positive for HMGB1. On the contrary, only a small fraction of CD3^+^ expressed HMGB1. Similar data were obtained analyzing PB cells (data not shown). In order to identify the various subsets of HMGB1 positive lymphocytes, the CD3^+^ HMGB1^+^ cell population has been further characterized by flow cytometry using monoclonal antibodies for CD4, CD8 and TCR gammadelta (Vδ2). As reported in Fig. 2C, cell surface expression of HMGB1 was confined primarily in Vδ2 T cells both in CB and PB. Also CD4^+^ T-cells resulted positive for HMGB1, whilst CD8^+^ lymphocytes did not present any expression of HMGB1.

**Figure 3:**
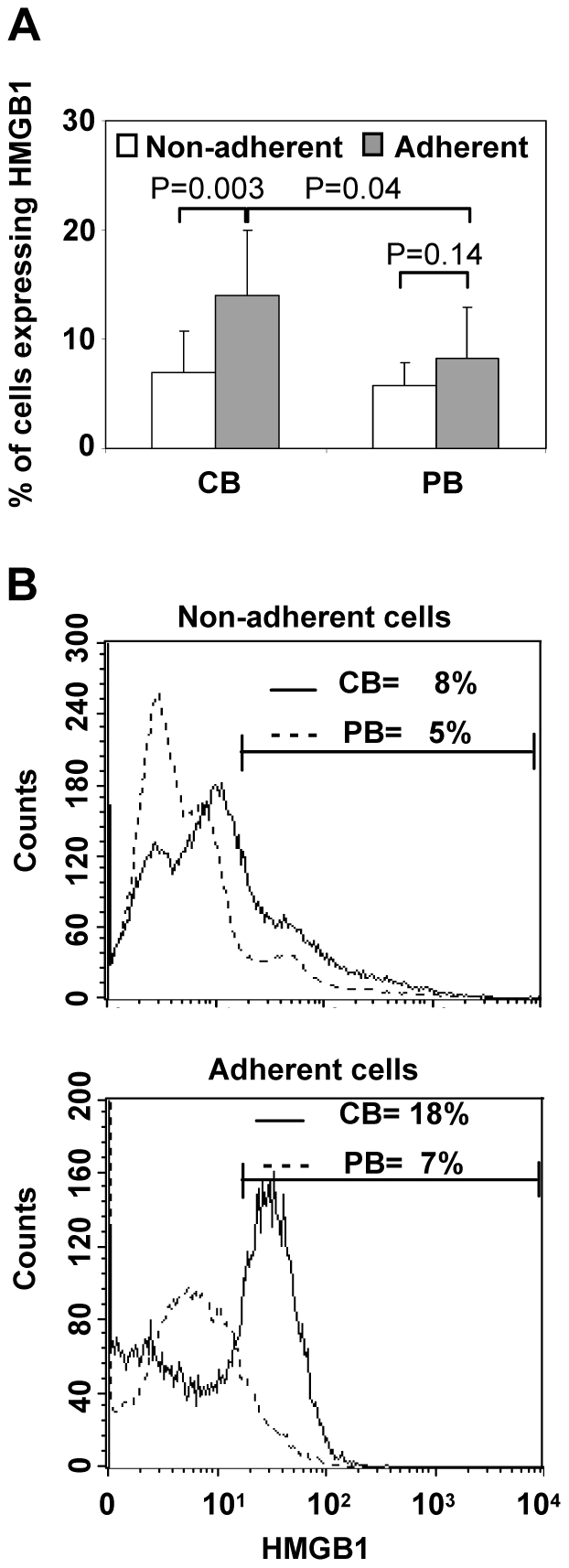
HMGB1 is present mostly in CB adherent cells. Mononuclear cells isolated from human CB and PB have been cultured in complete growth medium. After 14 days, two cell populations have been identified: adherent and non-adherent cells. (A) The cell surface expression of HMGB1 was determined in the two cell populations by flow cytometry analysis. Data are shown as percentage of cells expressing HMGB1 and values are mean ± SD of eight experiments from different donors. Statistical analysis compared non-adherent versus adherent cells or CB versus PB (*P<0.05, **P<0.01 paired Student's t test). (B) The fluorescence histogram plots displays HMGB1 levels in non-adherent and adherent CB (solid line) and PB cells (dotted line) and the percentage of HMGB1 positive cells is indicted in graphs. Data reported is representative of eight different experiments.

The previous reported data show the expression of HMGB1 on a population of differentiated cells having a high forward scatter, corresponding potentially to myeloid DC precursors. In order to further support them, we analyzed CB and PB cells after 14 days of cell culture because two different cell populations were identified: adherent and non-adherent cells. By FACS analysis, we observed that CB adherent cells expressed a significantly higher levels of HMGB1 than non-adherent (14%±5 versus 7%±3 in non-adherent cells, P = 0.003) (Fig. 3A–B). On the contrary, in PB the two cell populations displayed a similar levels of HMGB1 cell surface expression (P = 0.14). However, although not statistically significant, the PB adherent cells showed a trend toward higher HMGB1 expression compared to non-adherent cells. The different levels of HMGB1 expression between CB and PB cells observed at 48 h, has been found also after 14 days of culture in the adherent cell subset.

In this context, CB adherent cells shows a shift in the fluorescence curve to the right, indicating a greater expression of HMGB1 in adherent than non-adherent cells (Fig. 3B). On the contrary, in PB cells the level of fluorescence appears only slight different in adherent or non adherent subpopulations (Fig. 3B). Moreover, further characterization by FACS analysis indicated that HMGB1-positive adherent cells were CD14^+^CD11c^+^ and CD14^−^D11c^+^ cells, confirming the results obtained at 48 h (Fig. S1).

### Different stimuli modulate HMGB1 expression and its secretion

**Figure 4:**
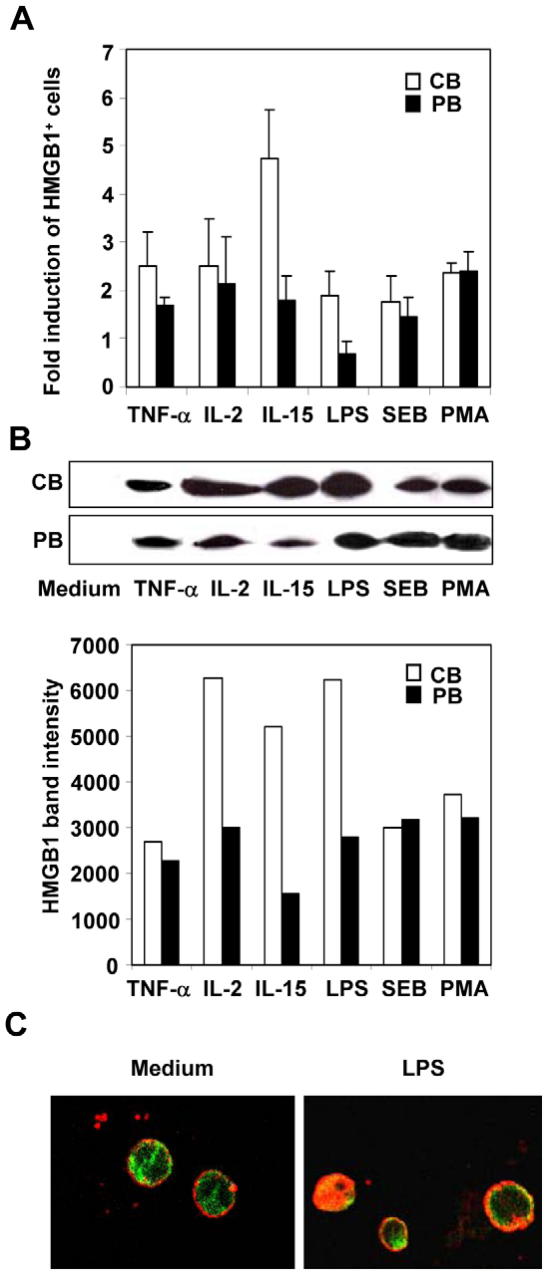
Different stimuli induce cell surface-expressed HMGB-1 and its secretion into extracellular environment. Various activation signals, such as proinflammatory stimuli (TNF-α or IL-2 or IL-15) or that mimic infection (LPS, SEB or PMA) have been used to influence the cell surface expression and active secretion of HMGB1. CB and PB cells have been treated with proinflammatory mediators for 14 days and stimuli that mimic infection for 48 h. (A) Cell surface expression of HMGB1 has been analyzed by FACS analysis, evaluating the percentage of positive cells. In bar graph the values are shown as ratio between treated and untreated cells expressing HMGB1 (fold induction). The values are the mean ± SD of four experiments from different donors. (B) The culture medium of CB or PB cells, which have been analyzed in (A) for surface HMGB1 expression, has been evaluated for its secretion by western blot analysis. The levels of protein shown in (B), quantified by densitometric analysis, has been expressed as arbitrary units. Western blot are representative of three independent experiments. (C) Untreated CB cells or treated with LPS for 48 h were fixed, permeabilized and stained with anti-HMGB1 antibody (green channel) and membrane-specific PKH26 red fluorescent dye. The fluorescence has been analyzed by confocal microscopy (Leica TCS SP5).

In peripheral blood, HMGB1 has been recently demonstrated to be a cytokine secreted by activated immune cells and mediate the response to infection, injury and inflammation. Therefore, we investigated whether various activation signals, such as proinflammatory stimuli (TNF-α or IL-2 or IL-15), or signals that mimic infection (LPS, SEB or PMA), influence the cell surface expression and active secretion of HMGB1 by human cord blood cells. As shown in Fig. 4A, flow cytometry analysis indicated that cell surface expression of HMGB1 was up-regulated by all stimuli. TNF-α increased HMGB1 levels by 2.5-folds in CB and 1.7-folds in PB cells over control at 14 days after treatment. At the same time point, up to approximately 5-folds increase in HMGB1 expression was observed in CB cells treated with IL-15 in contrast to PB cells in which up-regulation reached only 2-folds over control. In addition, IL-2 treatment showed a similar stimulation of HMGB1 expression in CB and PB cells. Similar trend in HMGB1 up-regulation has been observed after 48 h of treatment with stimuli mimicking infection. SEB and PMA determined a similar induction of HMGB1 expression both in cord blood and peripheral blood cells (2-folds), whilst LPS showed a higher increase of protein expression in CB (2-folds) than PB cells (0.8-folds). To determine whether the triggered expression of HMGB1 on cell membrane was associated to its secretion, western blot analysis was performed on the culture medium of CB and PB cells. All different stimuli induced secretion of HMGB1 from CB and PB cells at 48 h and 14 day after treatment (Fig. 4B), while no detectable amounts of HMGB1 has been found in untreated cell medium. Quantitative evaluation of HMGB1 band intensity revealed that IL-2, IL-15 and LPS determined a greater secretion of protein in CB than PB cells (Fig. 4B). On the other hand, TNF-α, SEB and PMA determined the secretion of similar amount of HMGB1 in the two cell types (Fig. 4B). Interestingly, the levels of cell surface expression and secretion of HMGB1 resulted strictly correlated. Moreover, the constitutive and the inducible expression level observed, presented a similar trend toward higher prevalence in CB cells.

To determine whether the modulation of HMGB1 expression in CB cells was associated to different intracellular localization of HMGB1, LPS treated CB cells were co-stained with anti-HMGB1 (stained in green) and membrane-specific PKH26 red fluorescent dye. Confocal immunofluorescence microscopy revealed that in resting CB cells HMGB1 presented heterogeneous labeling pattern, and was localized to the nucleus/cytoplasm, as well as under the apical membrane and faintly on cell surface (Fig. 4C). After forty-eight hours of LPS stimulation, HMGB1 appeared to move from the nucleus/cytoplasm, which is still partly positive, to the periphery of the cells and precisely around its external perimeter, as indicated by the colocalization with membrane-specific PKH26, well evident in the cell with peripheral section (Fig. 4C). This change in surface expressed HMGB1 was completely confirmed by FACS analysis above reported (Fig. 4A).

### The constitutive and inducible expression of HMGB1 is regulated via non-classical secretory pathway

**Figure 5:**
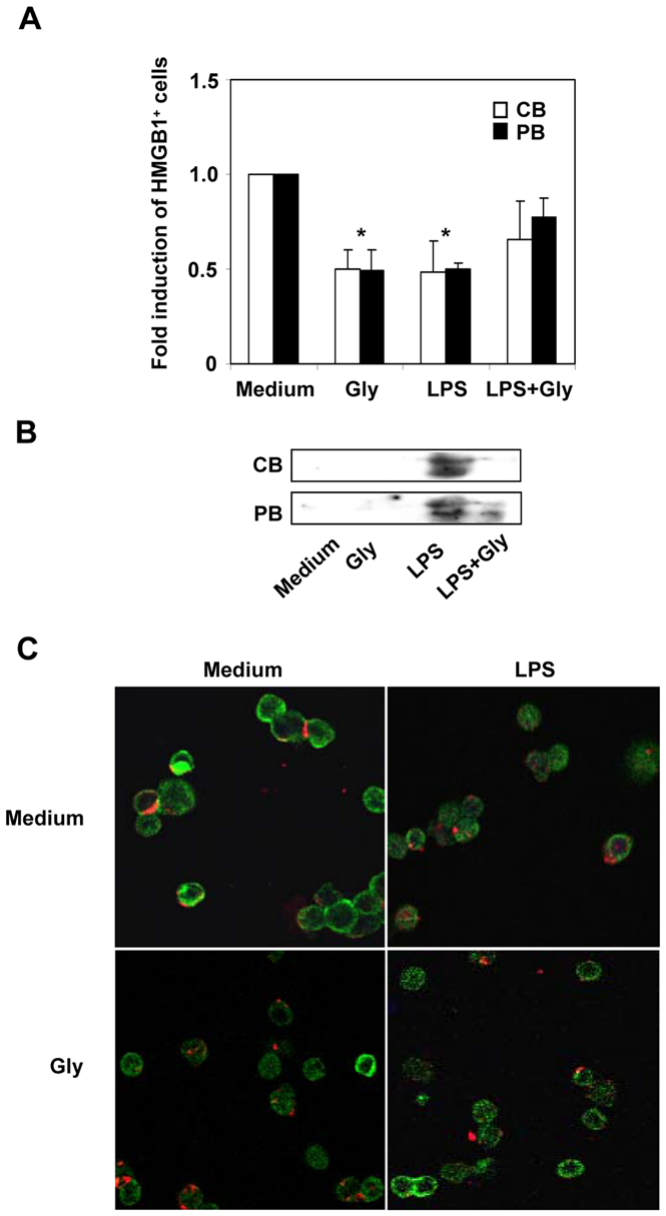
HMGB1 expression is impaired by inhibitor of the Atp binding cassette transporter ABC-1. To characterize the secretion pathway of HMGB1, mononuclear cells were treated with 100 µM glyburide, ABC-1 inhibitor, in presence or absence of LPS (0.5 µg/ml). (A) After 20 h, cell surface-expressed HMGB1 was evaluated by FACS analysis in CB and PB cells. The values are shown as ratio between treated and untreated cells expressing HMGB1 (fold induction). The values are the mean ± SD of three experiments from different donors. Statistical analysis compared treated versus untreated cells (*P<0.05 paired Student's t test). (B) The culture medium of CB or PB cells, which have been analyzed in (A), has been evaluated by western blot analysis to detect its secretion. Western blot is representative of three independent experiments. (C) CB cells were stained with anti-HMGB1 antibody (green channel) and membrane-specific PKH26 red fluorescent dye and analyzed by confocal mycroscopy. HMGB1 expression was evaluated in untreated and glyburide treated CB cells (Top and bottom left panel) or LPS and LPS plus glyburide treated CB cells (Top and bottom right panel) after 20 h of activation.

Recently, Gardella et al [29], showed that IL-1β and HMGB1 were secreted by monocytes via non-classical secretory pathway and HMGB1 secretion was reduced by Atp Binding Cassette trasporter (ABC-1) inhibitors. To investigate the effect of ABC-1 inhibitor on HMGB1 expression and secretion in CB and PB cells, mononuclear cells were treated with glyburide (100 µM) and/or LPS (0.5 µg/ml). After 20 h, we evaluated cell surface expression and release of HMGB1 by FACS analysis and western blot, respectively. The results showed that glyburide induced a 50% reduction in constitutive expression of HMGB1 on cell surface in CB and PB (P<0.05) (Fig. 5A). In addition, the ABC-1 inhibitor prevented the LPS effect at 20 h after treatment, partially restoring the constitutive cell surface expression of HMGB1 that resulted decreased as consequence of protein release induced by LPS (P>0.05). Western blot analysis indicated that glyburide blocked HMGB1 secretion induced by LPS in CB and PB cells (Fig. 5B). No cytotoxic effect was observed at 24 h after treatment with glyburide by MTT cytotoxicity assay. Altogether, these data suggests that the constitutive and inducible expression or secretion of HMGB1 is regulated through alternative, non-classical routes, as also confirmed by enhancing of its expression (2-folds over control) after Brefeldin A treatment (data not shown). The pharmacological modulation of HMGB1 expression was associated to different intracellular localization of HMGB1, as demonstrated by immunofluorescence analysis of CB cells co-stained with anti-HMGB1 (stained in green) and membrane-specific PKH26 red fluorescent dye (Fig. 5C). Confocal immunofluorescence microscopy revealed that in untreated CB cells HMGB1 was localized mainly under and on the apical membrane. Moreover, HGMB1 staining was observed to be concentrated at areas of cell-cell contact. In glyburide treatment HMGB1 became less concentrated and more dispersed resulting in a faint fluorescence, as quantified by FACS analysis. Moreover, the green fluorescence was often punctuate, suggesting cytoplasmic compartmentalization of the protein within vesicles. Twenty hours after stimulation with LPS, HMGB1 displayed a similar pattern of distribution observed after glyburide treatment, confirming the lower cellular expression of HMGB1 in treated than untreated cells observed by FACS analysis. Following the glyburide treatment, the modification of HMGB1 distribution induced by LPS was partially prevented, restoring a distribution of HMGB1 concentrated around the perimeter.

### HMGB1 is up-regulated by aminobisphosfonates

**Figure 6:**
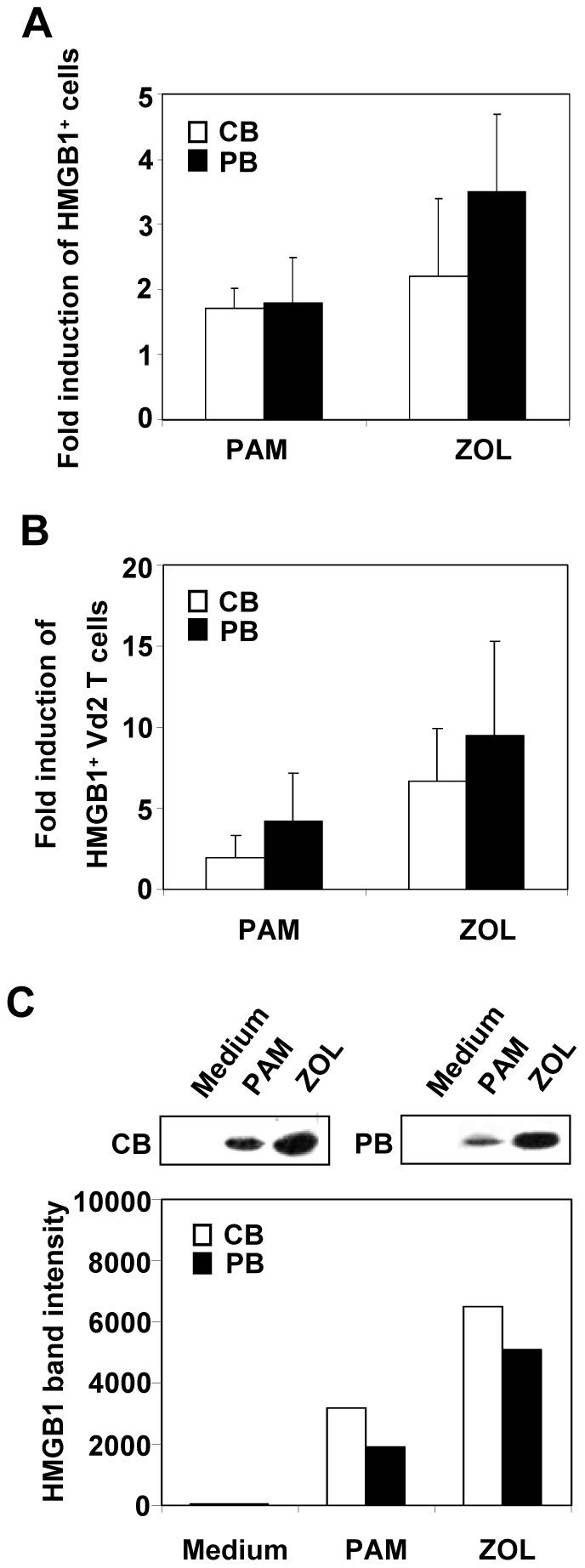
HMGB1 is up-regulated by Pamidronate and Zoledronate in CB and PB cells. Mononuclear cells isolated from CB and PB have been treated with Pamidronate (1 µg/ml) and Zoledronate (1 µg/ml). (A) After 14 days, cell surface expression of HMGB1 has been analyzed by FACS analysis and the values, shown as fold induction, are the product of ratio between treated and untreated cells expressing HMGB1. (B) By multi-color flow cytometric analysis, CB and PB cells have been gated for lymphocytes and stained with HMGB1, CD3 and gammadelta (Vδ2) antibodies. The ratio between treated and untreated HMGB1^+^CD3^+^ cells is shown as fold induction. The values reported are mean ± SD of four experiments from different donors. (C) The secretion of HMGB1 in cell culture medium was evaluated by western blot analysis. Densitometric analysis of western blot has been expressed as arbitrary units. Western blot is representative of three independent experiments.

Since HMGB1 was expressed on γδ T lymphocytes, we evaluated whether aminonobisphosphonate compounds (ABs), Pamidronate (PAM) and Zoledronate (ZOL), known to induce activation and proliferation of γδ T lymphocytes, were able to trigger HMGB1 expression. After 14 days of cell culture in presence of ABs (1 µg/ml), FACS analysis indicated that cell surface expressed HMGB1 was upregulated by PAM and ZOL, reaching 2.2- and 3.5-folds induction over control in CB and PB cells, respectively, after ZOL treatment (Fig. 6A). Furthermore, both compounds determined an evident expansion of HMGB1 positive γδ T cells which were markedly increased by ZOL treatment (10-folds over control) (Fig. 6B). This data was completely in agreement with evaluation of HMGB1 secretion by western blot analysis performed 14 days after treatment with 1 µg/ml ABs. Both ABs led to a significant induction of HMGB1 secretion into extracellular environment (Fig. 6C). As observed with cell surface expression, ZOL triggered a stronger release of HMGB1 by CB and PB cells than PAM treatment. Even if PAM showed to have a lower effect on upregulation of HMGB1 expression than ZOL, its efficacy on HMGB1 release was significantly increased compared to control. Finally, to demonstrate that ABs-induced release of HMGB1 was not determined by cell death, at 14 day of treatment we evaluated apoptosis/necrosis by flow cytometry analysis after Annexin V/Propidium Iodide staining. No significant change in Annexin V and Propidium Iodide positive cells was observed in ABs treatment in comparison to control.

### HMGB1 mediates migration of monocytes

**Figure 7:**
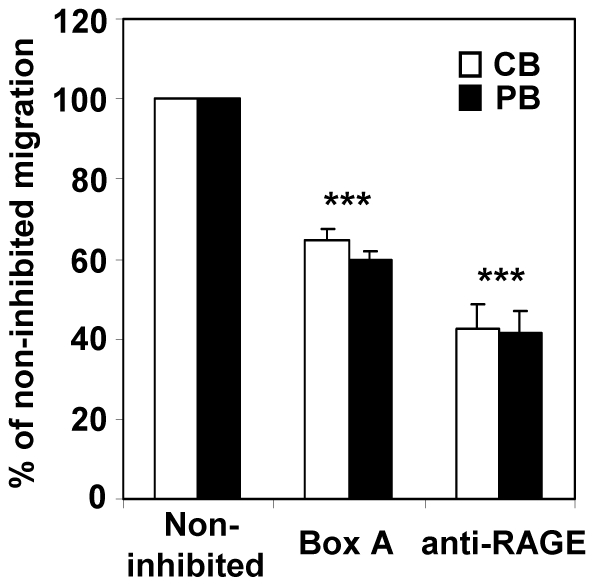
BoxA or anti-Rage antibody partially inhibits monocytes migration. Fresh mononuclear cells isolated from CB and PB have been added to upper well of transwell chamber to measure cell migration induced by pre-conditioned medium generated from the culture of CB or PB cells after 14 days of IL-2 treatment. The migration of monocytes (CD14^+^ cells) into lower chamber has been evaluated by FACS analysis. Noninhibited migration was defined as 100%. Bars represent mean ± SD of three different experiments. The inhibition of monocytes migration has been obtained by adding BoxA or anti-RAGE antibody in upper well of transwell chamber (***P<0.001).

Extracellular HMGB1 acts as immune-stimulatory signal that promotes recruitment of inflammatory cells, as monocytes and dendritic cells, by signaling through RAGE [30]. As we observed HMGB1 release after treatment with different stimuli, pre-conditioned medium generated from the culture of CB or PB cells after 14 days with IL-2 treatment, has been evaluated for its effect on cell migration. After 4 h pre-conditioned medium induced migration of CB or PB CD14^+^ monocytes through porous membrane. The phenomenon resulted inhibited by using N-terminal fragment of HMGB1, Box A (10 µg/ml), as HMGB1 antagonist [31], and anti-RAGE antibody (40 µg/ml) (Fig. 7). Moreover, the percentage of migrated cells resulted lower in presence of anti-RAGE than BoxA, indicating the presence of additional factors that binds RAGE in pre-conditioned medium. The HMGB1-mediated migration of monocytes has been further confirmed by using recombinant HMGB1 protein that induced a similar percentage of migrated cells as observed in pre-conditioned medium in presence of anti-RAGE antibody (data not shown). Finally, the secreted HMGB1 did not induce stem cells chemotaxis, because lacking RAGE expression, which is present on monocytes, as assessed by FACS analysis, confirming the inhibitory effect of anti-RAGE antibody on monocytes migration (data not shown).

## Discussion

High-mobility group box 1 has been isolated from calf thymus as an abundant nuclear protein over 30 years ago [32]. Recent studies demonstrated that HMGB1 is both actively secreted from activated leukocytes, as a late cytokine mediator [31], [33], [34] and passively released from necrotic or damaged cells [17]. Therefore, the released HMGB1 acts as trigger of inflammation, attracting inflammatory cells, and tissue repair in autocrine/paracrine fashion.

The importance of HMGB1 as inflammatory mediator examined in adult immune system has been also discovered in fetuses and newborns in which soluble RAGE and HMGB1 are active participants of the tissue injury process [27]. Moreover, in neonates with asphyxia it has been suggested that the elevation of HMGB1 might be associated with abnormal inflammatory responses involving the excessive production of proinflammatory cytokines [35]. The important role of HMGB1 in fetal immune system has been corroborated by data demonstrating that in response to stimuli HMGB1 secreted by human umbilical vein endothelial cells (HUVEC) triggers inflammatory responses through up-regulation of adhesion molecules and release of soluble proinflammatory mediators from endothelial cells [36], [37]. Fetal and newborn immune system is characterized to be phenotypically and functionally immature [38]. The innate immune system is the first line of defense against infections in neonates, providing critical protection before the generation of an appropriate adaptive immune response. In consideration of the essential role of neonatal immune responses and the important biological function of HMGB1 as bridge between innate and adaptative immune responses, here we have shown for the first time that HMGB1 is expressed and secreted from mononuclear cells isolated human cord blood. By FACS analysis, we demonstrated that CB cell surface membranes are positive to HMGB1 and its expression is significantly higher in adherent cells than non-adherent cells obtained after long term cell culture. These results show that constitutive HMGB1 expression is confined to a population of differentiated cells, likely myeloid DC precursors in CB and PB. Conversely, lymphocytes, as CD4 or γδ T cells, present a small cell fraction positive for HMGB1. These findings are completely in agreement with the role of HMGB1 in regulating immune response, in which activated monocytes and dendritic cells are the main source of HMGB1 release, promoting their functional maturation in autocrine/paracrine fashion and sustaining the proliferation and polarization of antigen-specific T-cells towards a Th1 phenotype (CD4). γδ T cells represent a small subset of T cells that possess a distinct T cell receptor (TCR) on their surface [39]. These cells exhibit several characteristics that place them, as HMGB1, at the border between the more evolutionarily primitive innate immune system and the adaptive immune system. Thus, our observations give further evidence to the important role of HMGB1 in innate immunity in which macrophages/monocytes, DC and γδ T cells are the main components. Vγ9Vδ2 T cells, which represent the major subset of circulating human γδ T cells, react against a set of non-peptidic, phosphorilated antigens recognized in a TCR-dependent manner [40]. These compounds derive from the mevalonate pathway which is essential for mammalian cells in the sterol synthesis, cell growth and membrane integrity. Other human γδ T cell antigens are synthetic compounds Aminobisphosphonate, such as Pamidronate and Zoledronate [41], known as potent inhibitors of osteoclast-mediated bone resorption used for the treatment of osteoporosis, bone metastasis and cancer [21], [42]. It has been shown that bisphosphonates exert a stimulatory effect on adult peripheral blood γδ T cells, *in vitro* and *in vivo*, by inhibiting the mevalonate pathway [43]–[45]. Recently, we have reported that the treatment with ABs induces proliferative responses in cord blood Vδ2 T cells accompanied by modifications of their naïve phenotype towards a regulatory subset, indicating that they are not inherently unresponsive [46], [47]. In this study, we demonstrated that PAM and ZOL trigger cell surfaced expressed HMGB1 in CB and PB cells with an evident increase of HMGB1 positive γδ T cells. Furthermore, ABs treatment leads to remarkable secretion of HMGB1 in extracellular environments. This study provide the first demonstration that ABs treatment modulate the expression of HMGB1 in CB cells, involving Vδ2 T-cells directly or throughout their presentation by APC cells (monocyte lineage) which modulate surface molecules or release cytokines needed for optimal Vδ2 T cell activation, as reported by Miyagawa F et al [48]. Moreover, the enhanced secretion of HMGB1 is not caused by increased apoptosis or necrosis, as demonstrated by FACS analysis. Different stimuli, and not only ABs, are able to modulate HMGB1 expression in CB. In our study, we demonstrated that stimuli that mimic infection (LPS, SEB or PMA) or pro-inflammatory mediators, as TNF-α or IL-2 or IL-15, induce the cell surface expression of HMGB1 and its secretion at 48 h or 14 days after treatment, respectively. Besides, the addition of glyburide, an ABC-1 inhibitor, inhibits LPS-induced secretion, indicating that HMGB1 is released by non-classical secretion pathway, as previously showed by Gardella S. et al [29]. Interestingly, the inhibition of HMGB1 secretion is strictly correlated to its cell surface expression, quantified by FACS analysis, in which ABC-1 inhibitor is able to reduce either its constitutive or inducible expression, demonstrating a role of cell membranes in HMGB1 secretion. As confirmed by confocal microscopy analysis, merged images of LPS treated CB cells verify the almost complete colocalization of HMGB1 and plasma membrane. Moreover, HGMB1 staining is often visualized punctuate, suggesting compartmentalization of the protein within cytoplasmic vesicles and is predominantly localized toward the apical ends, areas of cell-cell contact. Our data are in agreement with data presented by Beer Stolz D (Pittzburg, PA, USA) and Rouhiainen et al [49], suggesting that HMGB1 is actively secreted from cells by multivescicular endosomes fuse with plasma membrane or that monocytes/macrophages express at cell surface HMGB1, indicating HMGB1 as mediator of cell-to-cell or cell-to-matrix interaction to facilitate their recruitment by binding RAGE at endothelial cells [50].

Results presented herein provide a new insight into the role of HMGB1 in CB innate immune response. We demonstrate that stimuli, as LPS or cytokines or synthetic compounds, can initiate a cascade of events that lead to the activation of immune cells and secretion of mediators, as HMGB1. Once secreted into extracellular milieu, HMGB1 can function as a cytokine to contribute to infectious and inflammatory disorders, as confirmed by our data of its ability on CB monocytes recruitment, mediated by RAGE. Human umbilical vein endothelial cells release HMGB1 and express RAGE. Therefore, HMGB1, which is involved in a paracrine interaction, might play a crucial role in transendothelial migration and consequently in inflammatory immune response in CB.

Whereas a blockade of extracellular HMGB1 might represent a suitable therapeutic target for the treatment of sepsis, the development of the appropriate cell-mediated immunity, which is associated with a Th1 type immune response, is essential for successful immunization. Besides, extracellular HMGB1 has been shown to act as immune adjuvant by enhancing immunogenicity of apoptotic lymphoma cells and eliciting antibody responses to soluble ovalbumin protein [51]. Moreover, a short peptide, named Hp91, identified within the B box domain of HMGB1, induced activation of human and mouse DCs, increasing secretion of pro-inflammatory cytokines and chemokines, including the Th1 cytokine, IL-12 [52]. Therefore, these immunostimulatory properties make HMGB1 an attractive candidate as an adjuvant for vaccine development. The stimulation of neonatal response with vaccines has certain medical advantages and, namely, produce early protection for the vulnerable newborn period. Successful vaccines contain an adjuvant component that activates the innate immune system, thereby eliciting antigen-specific immune responses. Many adjuvants appear to be ligands for toll-like receptors (TLR), as HMGB1, which are promising targets for the development of novel adjuvants to elicit vaccine immunogenicity [53], [54]. In this scenario, aminobishoshponates that are able to stimulate innate immunity, as γδ T cells in CB [47], and to induce HMGB1 secretion may be an immuno-modulating tool to approach neonatal pathologies. Moreover, in adult counterpart, considering that ZOL or PAM exert also anti-cancer activity by inducing apoptosis, ABs, as other anticancer agents, may interfere in the complex interaction between tumor and host immune system by the release of inflammatory mediators, such as HMGB1, which mediate cross-presentation of tumor antigens via binding on TLR4 and the promotion of tumor specific cytotoxic T cell responses [55], [56]. In conclusion, by modulating the activity of HMGB1 we might provide a potential therapeutic target in adult and neonatal pathologies.

## Materials and Methods

### Ethics statement

Human umbilical cord blood and buffy coats from peripheral blood donations samples were collected after obtaining informed consent. The consent was written and approved from all participants. All blood samples were only collected from donors that had consented scientific use of blood products. Ethics Committee approval for this study is not required according to institutional guidelines. In particular, approval from the Ethics Committee of our institution was not necessary because blood samples were obtained in compliance with Italian legislation and donors gave informed written consent to donation for research purpose in case of sample with cell content numerically unsuited for clinical use, and therefore are considered as residual sample or waste material. Informed written consent has been requested to the donors or to the authorized parents.

### Preparation of cells and culture conditions

Cord blood (CB) was obtained from healthy mothers according to institutional guidelines. Anonymous buffy coats from peripheral blood (PB) donations were collected from healthy blood bank donors. CB samples were obtained from spontaneous partum and normal full-term pregnancies by venipuncture of umbilical vein immediately after delivery. Samples were collected at S. Pietro Fatebenefratelli hospital in Rome. Cord blood was diluted (1∶1) with phosphate-buffered saline, PBS (Dulbecco's Phosphate Buffer Saline), and further diluted (1∶1) with a solution of 4% dextran in PBS (Sigma Aldrich, St Louis, USA). After sedimentation of erythrocytes, CB and PB samples was layered over Ficoll-Hypaque (Sigma-Aldrich, St. Louis, USA) density gradient and centrifugated at 1,800 rpm for 20 minutes. Freshly isolated mononuclear cells of CB and PB were cultured at 3×10^6^/ml in RPMI 1640 (Sigma Aldrich) supplemented with 10% FBS (Sigma Aldrich), 2 mM L-glutamine (Sigma Aldrich), 10 UI/ml penicillin-streptomycin (Sigma Aldrich).

HeLa cells (ATCC CCL-2) human cervical carcinoma, were cultures in DMEM (Sigma Aldrich) supplemented with 10% FBS (Sigma Aldrich), 2 mM L-glutamine (Sigma Aldrich), 10 UI/ml penicillin-streptomycin (Sigma Aldrich).

### Cell treatment

Fresh CB and PB cells were treated with the following stimuli: IL-2 (20 ng/ml) (Roche), TNF-α (20 ng/ml) (eBioscience, Inc), IL-12 (20 ng/ml) (eBioscience, Inc) or aminobisphosphonates, Pamidronate and Zoledronate (Zometa, Novartis Pharmaceutical) (1 µg/ml) for 14 days; Lipopolysaccharide (LPS) (500 ng/ml) (Sigma Aldrich), Staphylococcus aureus Enterotoxin B (SEB) (1 µg/ml) (Sigma Aldrich) or Phorbol 12-myristate 13-acetate (PMA) (30 ng/ml) (Sigma Aldrich) for 48 h. Untreated mononuclear cells have been cultured in complete growth medium for 48 h and 14 days. For secreted HMGB1 detection, culture supernatants were microcentrifuged at 1200×g for 5 minutes and frozen at −70°C until analysis.

### Flow Cytometric Analysis of Surface-Expressed HMGB-1 and Expression of cellular differentiation markers

Surface expressed HMGB1 has been analyzed by flow cytometry (FACSCalibur Flow Cytometry System, BD BioSciences Pharmingen) using the anti rabbit-HMGB1 antibody (Sigma Aldrich) and secondary FITC-conjugated antibody (BD Biosciences) or Alexafluor 647–conjugated antibody (Molecular Probes, Invitrogen). The surface expression of cell differentiation markers have been evaluated with the following fluorescently-conjugated antibodies: PE anti-human CD14 (M5E2), PE-Cy5 anti-human CD11c (B-ly6), PE-Cy7 anti-human CD16 (3G8), PE anti-human Vδ2 (B6), PE anti-human CD8 (SK1), FITC anti-human CD4 (RPA-T4) (BD Biosciences, PharMingen) and APC anti-humam CD3 (UCHT1) (eBioscience, Inc). The fluorescence labelling has been performed by incubating cells at 4°C (protected from light) in PBS with 4% bovine serum albumin for 30 minutes with antibodies. Cells were subsequently washed, resuspended in 500 µl PBS and acquired on flow cytometer (FACSCalibur, BD Biosciences). 100000 events have been collected and analyzed by Cell Quest program (BD Biosciences).

For Intracellular staining of HMGB1, CB and PB cells were fixed and permeabilzed with Cytofix/Cytoperm solution (BD Cytofix/Cytoperm Kit, BD BioSciences Pharmingen) prior to the addition of anti-HMGB1 antibody.

### Western Blot Analysis of Secreted HMGB1

CB and PB cell culture supernatants or complete culture medium (100 µl) were boiled in reducing Laemmli sample buffer, resolved on 12% SDS/PAGE under reducing conditions and electrotransferred onto PVDF filters (Hybond-P, Amersham Pharmacia Biotech, Milan, Italy), which were stained with Ponceau S (Sigma) and de-stained prior to blocking with 5% non-fat dry milk in PBS containing 0.05% Tween (Sigma) for 1 h. Filters were stained with polyclonal rabbit anti-HMGB1 antibody overnight at 4°C followed by anti-rabbit IgG horse-radish-peroxidase-(HRP-) conjugated secondary antibody for 1 hour at room temperature. HMGB1 detection was performed using Super Signal substrate (Pierce, Rockford, IL) according to the manufacturer's instructions. Quantitative evaluation of HMGB1 protein was determined by densitometric analysis.

### Inhibition of HMGB1 expression and secretion by ABC-1 inhibitor glyburide

In order to characterized the secretion pathway of HMGB1, CB and PB mononuclear cells (3×10^6^ cells/ml) have been activated with LPS (500 ng/mL) in presence of 100 µM glyburide (Sigma), a potent inhibitor of the secretion via Atp binding cassette transporter (ABC-1). After 20 hours HMGB1 has been evaluated as cell surface expression and its secretion in the culture medium by FACS and western blot analysis, respectively, as indicated before. The potential cell toxic effects of glyburide and its solvent (dymethylsulfoxide, DMSO) were determined by MTT.

### Confocal fluorescence microscopy

CB and PB mononuclear cells (3×10^6^ cells/ml) have been deposited on glass slides by centrifugation at 400 rpm for 5 minutes using a cytospin system (Thermo Shandon, Pittsburgh, PA). They were air dried, fixed in cold 4% paraformaldehyde for 15 minutes. Cells were washed in PBS and permeabilized in 0.2% Triton X-100 in PBS for 10 min at 4°C and then blocked with 1% bovine serum albumin in PBS for 40 min. Immunofluorescence staining of cells was performed using rabbit anti-HMGB1 antibody (1∶300) and anti-rabbit-FITC conjugated secondary antibody (1∶200). Antibodies dilutions in PBS containing 0.1%BSA were added to cells and incubated for 1 h at room temperature. After washing in PBS, general cell membrane labeling PKH26-GL red fluorescent dye (1∶250 dilution; Sigma) was added for 10 min at room temperature. Cells were washed three times in PBS, mounted using ProLong Gold antifade reagent (Molecular Probes, Invitrogen) and then analyzed using confocal microscope (Leica TCS SP5). Software: LAS AF version 1.6.3 (Leica Microsystem).

### MTT assay

Cytotoxicity has been quantified by measurement of the reduction of MTT (3-(4,5-Dimethylthiazol-2-yl)-2,5- diphenyltetrazolium bromide, a tetrazole) (Promega) to produce a dark blue formazan product. MTT has been added to each well 20 h after the beginning of the insult. After 3 h incubation, the Solubilization/Stop Solution has been added to the culture wells to solubilize the formazan product, and the absorbance at 570 nm recorded using a 96-well plate reader (Bio-Rad® Laboratories)

### Detection of apoptosis

CB and PB mononuclear cells (1×10^6^ cells/mL) were washed with ice-cold PBS and the cell pellets were resuspended in ice-cold binding buffer. Five µL Annexin V FITC solution and 5 µL propidium iodide (Bender MedSystems, Austria) were added to 490 µL of the prepared cell suspension and incubated at 4°C for 10 min in the dark. Aliquots were directly aspirated into a FACSCalibur flow cytometer and apoptosis analysed by CellQuest program.

### Monocytes chemotaxis assay

Fresh mononuclear cells isolated from CB or PB were placed to upper compartment of the Transwell chamber (3-µm pore size) and pre–conditioned medium, generated from the culture of CB or PB cells after 14 days of treatment and centrifugation at 1200×g, was added in lower well. After 4 h, migrated cells were characterized by FACS analysis. BoxA (HMGBiotech, Milan, Italy) or anti-RAGE antibody (Millipore, USA) were used to inhibit HMGB1 mediated cell migration.

### Statistical Analysis

Student's *t*-test (one-tail) has been used to assess the significance of differences in HMGB1 expression. Differences were considered significant if the probability of the null hypothesis was less than five percent (* *P*<0.05, ** *P*<0.01, *** *P*<0.001).

## Supporting Information

Figure S1 **Adherent cells are myeloid DC precursors expressing HMGB1.** At 14 days after isolation from CB and PB, adherent mononuclear cells were characterized by multi-color flow cytometric analysis of surface-expressed HMGB1 and expression of cellular differentiation markers. CD14^+^CD11c^+^ and CD14^−^ CD11c^+^ phenotypes are adherent cells expressing HMGB1. Values (mean ± SD of five experiments from different donors) are expressed as percentage of HMGB1 positive cell subset among the totality of HMGB1 expressing cells.
(TIF)
