# Supplementary figures and images for: HMGB1 and Cord Blood: Its Role as Immuno-Adjuvant Factor in Innate Immunity
Source: PLoS One. 2011 Aug 22;6(8):e23766. doi: 10.1371/journal.pone.0023766 (PMC3161821; doi:10.1371/journal.pone.0023766)

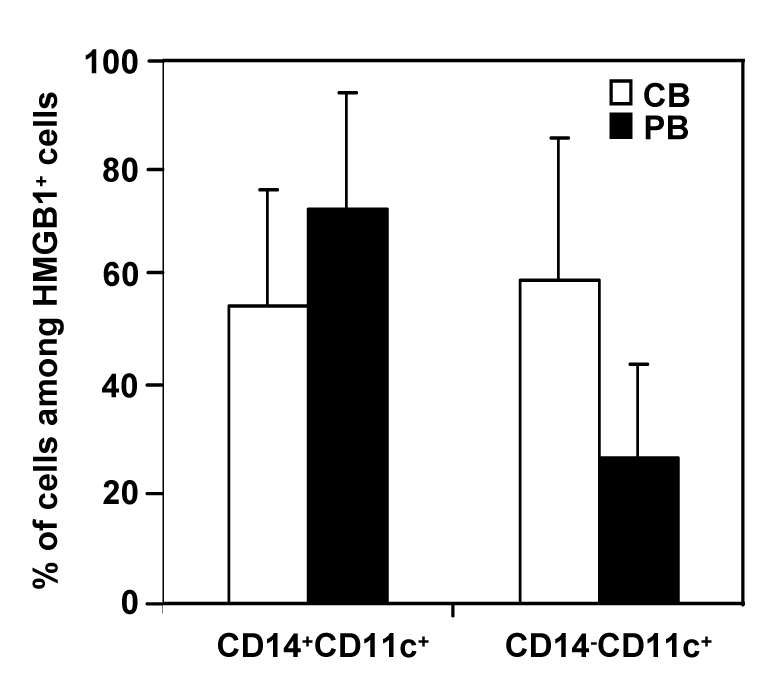

Supplement: Figure S1 — Adherent cells are myeloid DC precursors expressing HMGB1. At 14 days after isolation from CB and PB, adherent mononuclear cells were characterized by multi-color flow cytometric analysis of surface-expressed HMGB1 and expression of cellular differentiation markers. CD14+CD11c+ and CD14− CD11c+ phenotypes are adherent cells expressing HMGB1. Values (mean ± SD of five experiments from different donors) are expressed as percentage of HMGB1 positive cell subset among the totality of HMGB1 expressing cells. (TIF) [file pone.0023766.s001.tif]
